# Association of phosphorylation status of ERK and genetic MAPK alterations in pediatric tumors

**DOI:** 10.1038/s41598-025-98514-x

**Published:** 2025-04-18

**Authors:** Florian Selt, Romain Sigaud, Andrey Korshunov, David Capper, David Reuss, Andreas von Deimling, Kristian W. Pajtler, Cornelis M. van Tilburg, Martina Nesper-Brock, David T. W. Jones, Stefan M. Pfister, Felix Sahm, Olaf Witt, Till Milde, Jonas Ecker

**Affiliations:** 1 Hopp Children’s Cancer Center Heidelberg (KiTZ), Im Neuenheimer Feld 430, 69120 Heidelberg, Germany; 2 Clinical Cooperation Unit Pediatric Oncology, German Cancer Research Center (DKFZ) and German Consortium for Translational Cancer Research (DKTK), Heidelberg, Germany; 3 Clinical Trial Unit (ZIPO), Department of Pediatric Hematology, Oncology, Immunology and Pulmonology, Heidelberg University Hospital, Heidelberg, Germany; 4 National Center for Tumor Diseases (NCT), Heidelberg, Germany; 5 Department of Pediatrics and Adolescent Medicine, University Hospital Jena, Friedrich Schiller University Jena, Jena, Germany; 6 Comprehensive Cancer Center Central Germany (CCCG), Jena, Germany; 7 Department of Neuropathology, Heidelberg University Hospital, Heidelberg, Germany; 8 Clinical Cooperation Unit Neuropathology, German Cancer Research Center (DKFZ) and German Consortium for Translational Cancer Research (DKTK), Heidelberg, Germany; 9 Department of Neuropathology, Charité – Universitätsmedizin Berlin, Campus Charité Mitte, German Consortium for Translational Cancer Research (DKTK), Berlin, Germany; 10 Division of Pediatric Neurooncology, German Cancer Research Center (DKFZ) and German Consortium for Translational Cancer Research (DKTK), Heidelberg, Germany; 11 German Cancer Research Center (DKFZ), Heidelberg, Germany; 12 Pediatric Glioma Research, German Cancer Research Center (DKFZ) and German Consortium for Translational Cancer Research (DKTK), Heidelberg, Germany

**Keywords:** Relapsed pediatric tumors, Molecular diagnostics, MAPK alterations, pERK, Targeted therapy, pERK H-score, Cancer, Oncology

## Abstract

The mitogen-activated protein kinase (MAPK) pathway is one of the most frequently altered pathways in pediatric cancer. Activating genomic MAPK-alterations and phosphorylation of the MAPK downstream target ERK (pERK) were analyzed in the PTT2.0 registry to identify potential targets for MAPK-directed treatment in relapsed pediatric CNS tumors, sarcomas and other solid tumors. The present study investigates the association of ERK phosphorylation and genomic MAPK pathway alterations (mutations, fusions, amplifications) in the PTT2.0 dataset. PTT2.0 registry cases with available genomic and immunohistochemistry data (*n* = 235) were included. Samples with and without detected activating genomic MAPK alterations were compared regarding ERK phosphorylation, quantified by immunohistochemistry H-score. The association of pERK intensity and the presence of MAPK alteration was analyzed using a univariable binary logistic regression model.The mean pERK H-score was significantly higher in samples with activating genomic MAPK alterations. pERK H-score positively correlated with the presence of MAPK alterations. However, the pERK H-score predicted MAPK alterations only with a sensitivity of 58.3% and a specificity of 83.8%. The highest mean pERK H-scores were observed in low-grade gliomas, enriched for MAPK alterations, and in ependymoma, where MAPK alterations were absent. Although there is an association between pERK level and activating genetic MAPK alterations, the predictive power of pERK H-score for genetic MAPK alterations is low in pediatric tumors. Tumors/groups with absent genetic MAPK alterations but high pERK indicate a dissociation of the two parameters, as well as a possible MAPK pathway activation in the absence of genetic MAPK alterations.

## Introduction

The era of molecular diagnostics paves the way towards detecting actionable targets in pediatric tumors and potentially opens new avenues towards successful therapies for patients with dismal prognosis. Genetic alterations within the MAPK pathway leading to pathway activation are frequently found in pediatric cancers^1^ rendering the activated MAPK pathway one of the most abundant potentially actionable targets in pediatric cancer. Several studies have deomonstrated response of pLGG and other pediatric cancer types with genetic alterations in the MAPK pathway to treatment with MAPKi. The combination therapy consisting of the RAF inhibitor dabrafenib and the MEK inhibitor trametinib was approved for BRAF V600E positive gliomas^2^. Following a phase II trial in relapsed patients, the BRAF inhibitor (BRAFi) tovorafenib has been approved by the FDA for the treatment of relapsed or refractory MAPK-altered pLGG^3,4^ and is currently being tested against standard of care chemotherapy within the LOGGIC/FIREFLY-2 phase III study^5^. The INFORM registry showed that BRAF alterations are overrepresented in the very high-level evidence target group and survival analyses showed that BRAFi treatment improved the survival in patients with tumors harboring BRAF alterations^6–8^. This data highlights the significance of activating genetic MAPK alteration as biomarker for response prediction of MAPK-targeted treatment approaches in pediatric cancers. The PTT2.0 registry, which included patients with relapsed pediatric CNS tumors, sarcomas and other solid tumors, detected phospho-levels of the MAPK downstream effector ERK (pERK) as “functional” marker for MAPK pathway activation in addition to genomic MAPK alterations^9^. However, it is still unknown how well these two parameters are correlated. Therefore, the present study investigates, if the two parameters “genomic MAPK alteration” and “pERK levels” are associated/correlated in the setting of relapsed pediatric cancers. To this aim, data of the PTT2.0 registry study was evaluated.

## Materials and methods

### Tumor samples

The present study analyzed data obtained from FFPE tumor samples of the PTT2.0 registry study cohort, which was described in Ecker et al.^9^. In short, 263 patients with relapse or progression of their tumor were included. 178 (68%) had CNS tumors, 41 (16%) sarcomas and 44 (17%) other solid tumors. The median age of all patients included was 10 years. The FFPE tumor material analyzed originated from primary disease in 42% and from the current relapse episode in 57% of cases. As described before the registry was approved by the ethic committee of the Medical Faculty of the University of Heidelberg, Germany (S-546/2016; German Clinical Trial Register number: DRKS00011707) and all methods were carried out in accordance with all relevant guidelines and regulations. Written informed consent was obtained from all patients/legal guardians before inclusion into the registry^9^.

### Histopathological and molecular diagnostics

Molecular tumor classification and detection of genomic tumor alterations (single nucleotide variants (SNVs), gene fusions, amplifications) were performed within the PTT2.0 registry^9^. A detailed description of the immunohistochemistry, representative images showing the appearance of the stainings and the calculation of H-scores can be found in Selt et al.^9^. In brief, at least two experienced neuropathologists of the department of Neuropathology, Heidelberg evaluated the stained slides and calculated the pERK H-Scores based on the staining intensity of only the tumor cells.The H-score was obtained by the formula: H‐Score = 3 × percentage of strongly staining nuclei (%IHC3+ ×3) + 2 × percentage of moderately staining nuclei (%IHC2+ ×2) + percentage of weakly staining nuclei (%IHC1+ ×1), with a resulting range of 0–300.

### Statistical analyses and graphics

Comparisons between multiple groups were performed using One-way ANOVA and Tukey post-hoc test. Computational analyses and data visualization were performed using R Studio (R Version 4.1.0) as described before^10^. Logistic regression analysis was performed in R with the *glm* function as previously described^11^. Genomic alteration (present vs. absent) was the binary dependent variable, and phosphorylated ERK H-score was the independent continuous predictor used to generate the logistic regression model. A ROC analysis with varying H-score was performed to identify the best threshold discriminating between MAPK altered and non altered tumors, using the Youden’s J statistic. Finally, model performance for the identified threshold was assessed using sensitivity and specificity.

## Results

### Abundance of genomic MAPK-alterations and overall phosphorylation status of ERK

**Fig. 1:**
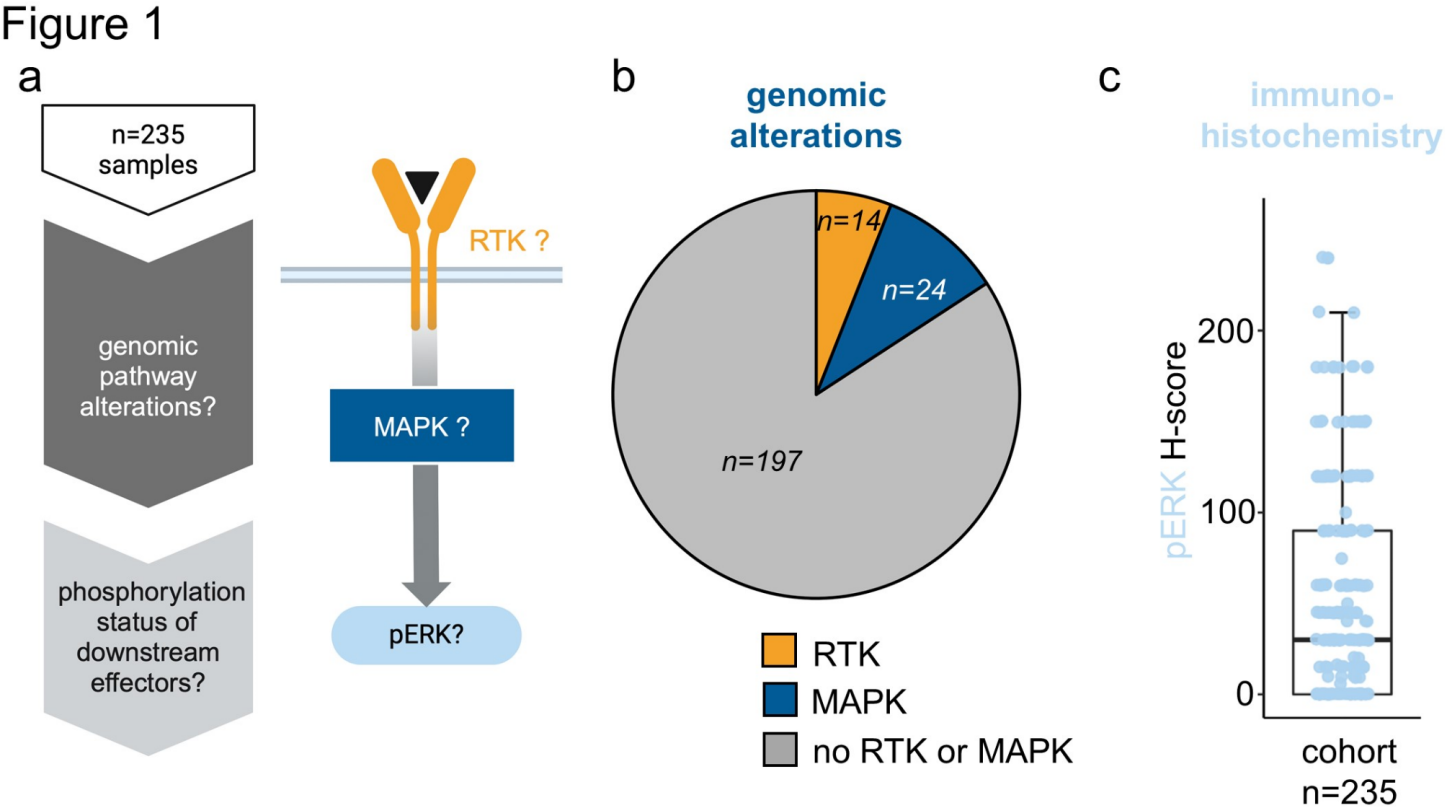
Abundance of genomic MAPK-alterations and phosphorylation status of ERK (n = 235 samples): (**a**) Depiction of the sample filtering workflow. (**b**) Pie chart depicting the fractions of the cohort with genomic BRAF alteration (blue), receptor tyrosine kinase alteration (RTK) (yellow) and no MAPK orRTK alteration (grey). (**c**) Boxplot depicting the distribution of pERK H-scores of all samples of the cohort. The Boxplots visualizes the minimum value, the first quartile (25th percentile), the median, the third quartile (75th percentile) and the maximum value.

The characteristics of the PTT2.0 cohort (*n* = 263 cases total; *n* = 178 (68%) CNS tumors; *n* = 41 (16%) sarcomas; *n* = 44 (17%) other solid tumors) including a detailed description of the genetic alterations can be found in Ecker et al.^9^. All cases of this cohort with available gene panel sequencing data, DNA methylation data and immunohistochemistry (IHC) data (*n* = 235) were included in the present study. The samples were screened for the presence of genetic alterations in the two categories: (1) activating alterations at the level of tyrosine kinases upstream of the MAPK pathway (including *PDGFR*, *FGFR* and *EGFR* alterations) and (2) activating alterations in the MAPK pathway (MAPK) (including *RAS*, *RAF* and *NF1* alterations) (Fig. 1a; Table 1). *n* = 14 samples (6%) had only upstream RTK alterations without MAPK alteration (yellow), *n* = 24 samples (10%) harbored an alteration in the MAPK pathway (blue) and no sample showed simultaneous alterations in both categories (Fig. 1b). As described by Ecker et al., the most frequent alterations in the group without MAPK or RTK alteration (Fig. 1b; grey) were related to the pathways phosphoinositide 3-kinase (PI3K), DNA repair/cell cycle, epigenetics and transcriptional regulation. The most commonly detected SNVs in this group were found within the genes *TP53* (10%), *ARID1A* (3%), *TERT* (3%), *CTNNB1* (2%), *FGFR1* (2%) and *SMARCB1* (2%). The most commonly found copy number variations were *CDKN2A/B* loss (8%),*TP53* loss (7%), *MYC* amplification (3%), *MDM2* amplification (3%) and *CDK4* amplification (2%)^9^. The median H-score for the phosphorylation status of the MAPK pathway downstream effector ERK in the cohort was 30, with a range of 0–240 (Fig. 1c).

**Table 1:** Description and distribution of the activating RTK and MAPK alterations of the cohort.

RTK alteration	Number of cases (total *n* = 14)
EGFR amplification	1
EGFR A289V	2
EGFR A289T	1
EGFR S1036T	1
FGF1 V472M + FGF1 N457K	1
FGFR1 K567E + FGFR1 A475V	1
FGFR4 V550L	1
FGFR4 V550M	1
FGFR4 G636C	1
PDGFRA amplification	3
PDGFRA G313R	1

MAPK alteration	Number of cases (total *n* = 24)
NF1 F934fs + NF1 loss	1
KRAS Q61L	1
KRAS Q22K + KRAS L19F	1
NRAS Q61K	1
NRAS Q61L	1
KIAA1549::BRAF-fusion	11
BRAF amplification	1
FAM131B::BRAF-fusion	1
SRGAP3::RAF1-fusion	1
BRAF V600E	5

### Association of genomic MAPK alterations and phosphorylation status of ERK

**Fig. 2:**
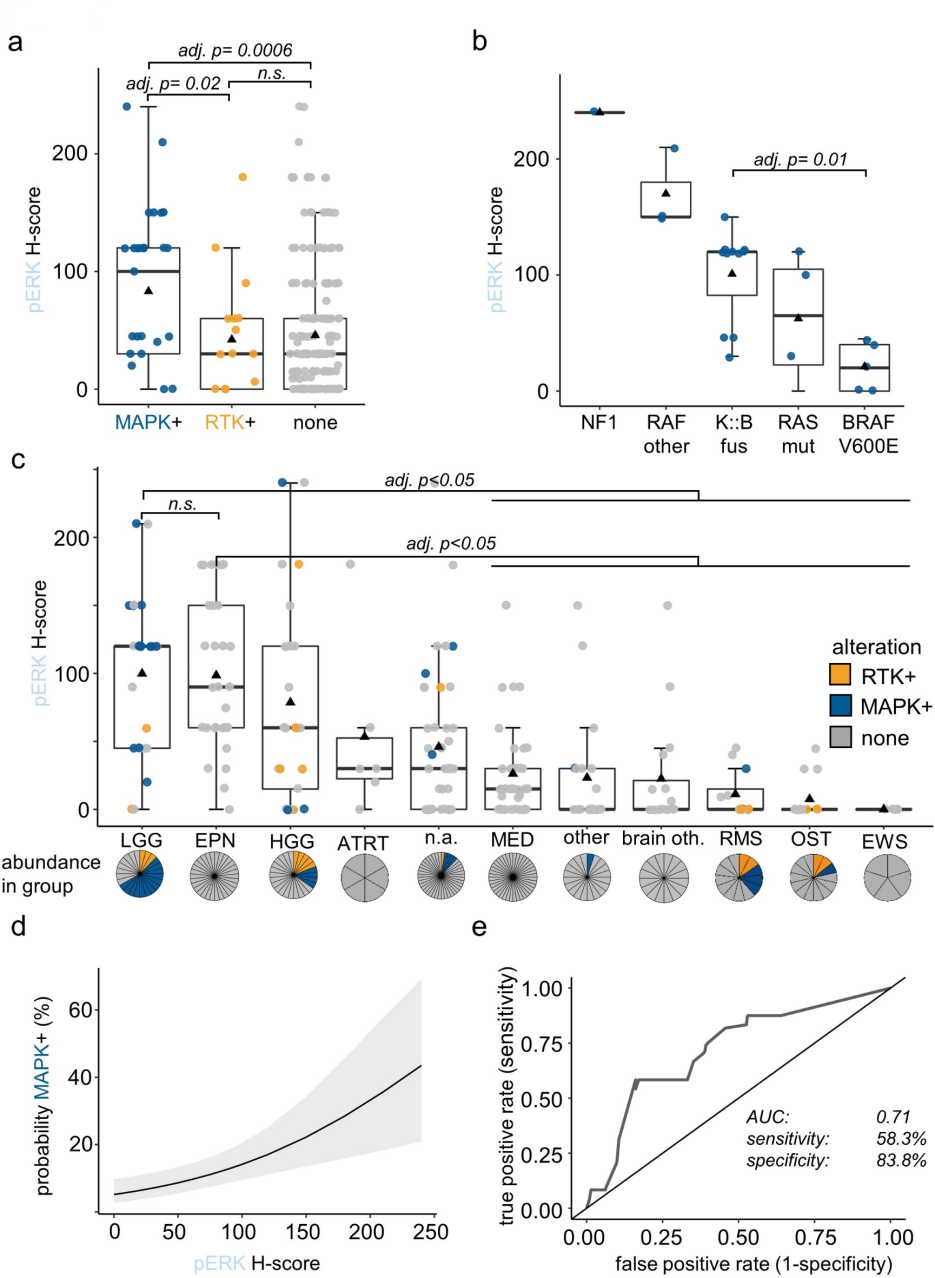
Association of genomic MAPK alterations and phosphorylation status of downstream effectors: Boxplots visualize the minimum value, the first quartile (25th percentile), the median, the third quartile (75th percentile) and the maximum value; black triangles indicate the mean (**a**) Boxplot comparing the pERK H-scores in the samples with MAPK alteration (MAPK+; blue) to samples with only RTK alteration (RTK+; yellow) and samples without alteration related to MAPK signaling (none; grey); n.s.: not significant; adj.*p*: adjusted *p*-value. (**b**) Boxplot comparing the pERK H-scores in samples with different MAPK alterations. NF1: *NF1* alterations; RAFother: structural *RAF* alterations other than *KIAA1549::BRAF* fusion, K::B fus: *KIAA1549::BRAF* fusion; RAS mut: *KRAS* and *NRAS* point mutations; BRAF V600E: *BRAF V600E* point mutations. adj.*p*: adjusted *p*-value. (**c**) Boxplot depicting the pERK H-scores in different tumor groups of the cohort. LGG: low-grade glioma, EPN: ependymoma; HGG: high-grade glioma; ATRT: atypical teratoid rhabdoid tumors, n.a.: samples without definitive methylation based classification; MED: medulloblastoma; other: tumors form other molecular classes outside the CNS; other brain: CNS tumors form other molecular classes than LGG, HGG, ATRT or MED; RMS: rhabdomyosarcoma; OST: osteosarcoma; EWS: En.s.: not significant; adj.*p*: adjusted *p*-value. The pie charts below the boxes indicate the abundance of alterations in the respective subgroups. (**d**) Probability of the presence of a MAPK alteration in relation to pERK level based on binary logistic regression analysis. Grey field indicates the 95% confidence interval. (**e**) ROC curve for sensitivity and specificity of pERK as predictor for the presence of a MAPK-alteration at an optimal pERK H-score cutoff of 91–99. AUC: Area under the curve.

Next the association between the presence of a MAPK alteration and pERK staining was investigated. The mean pERK H-score in samples with alteration in the MAPK pathway was significantly higher compared to samples without alterations in MAPK pathway or samples harboring RTK alterations. Samples with alterations in RTK were not significantly different from samples without alterations (Fig. 2a). Within the group of samples with MAPK alterations, the highest pERK H-score was observed in a patient with NF1 (H-score 240) and MAPK-altered tumors with *BRAF V600E* mutations had the lowest pERK H-scores (median 20; 0–45). The pERK levels in tumors with *KIAA1549::BRAF* fusions were significantly higher compared to *BRAF V600E* positive cases (Fig. 2b). When the samples were analyzed separated by entities the highest pERK H-scores were observed in the groups of low-grade gliomas (LGG) and ependymomas (EPN) (Fig. 2c). While the LGG samples, as expected, harbored a high proportion of MAPK alterations (54%), MAPK alterations were absent in the group of EPN. Mean pERK H-scores of LGG and EPN were significantly higher compared to medulloblastoma (MED), rhabdomyosarcoma (RMS), osteosarcoma (OST), ewing sarcoma (EWS) and other less frequent tumors in and outside of the CNS (“other” and “other brain”, respectively).

The ability of pERK staining to predict MAPK alteration was then evaluated. In a binary logistic regression analysis, the pERK H-score was significantly positively associated with the presence of a MAPK alteration (*p* < 0.01). The relationship between the pERK H-score and the probability of having a MAPK alteration was quantified by calculating the odds ratio. With an odds ratio of 1.01 the chance of having a MAPK alteration increases by 1% for 1 point in H-score. The predicted probabilities of having a MAPK-alteration based on pERK H-score is depicted in Fig. 2d. Based on the results of a ROC analysis, the sensitivity and specificity of pERK predicting a MAPK alteration was 58.3% and 83.8%, respectively, at an optimal H-score cut-off of 91–99 (Fig. 2e). Taken together, these data suggest that, while MAPK altered samples had a significantly higher pERK than non-MAPK altered, pERK positivity occurs in samples/entities without a driving MAPK alteration, making it a poor predictive marker for genetic MAPK alterations.

## Discussion

The MAPK pathway is among the most frequently altered potentially druggable pathways in a retrospective analysis of a pan-cancer cohort including 961 pediatric tumors^1^. In line with this study, the abundance of MAPK alterations in the PTT2.0 cohort was 10%. pLGG was the group with the highest abundance of MAPK alterations as expected^1,12^. However, the frequency of alterations (13% on the level of RTK and 54% in the MAPK pathway downstream of RTK; 67% in total) was lower than expected for this group^12,13^, although DNA methylation analysis allowed for a unambiguous classification of the samples as pLGGs. The methods applied for the detection of MAPK alterations in our registry (gene panel sequencing, DNA methylation and RNA sequencing in a limited number of cases) are not fully comprehensive and might have missed some MAPK alterations. For example, some relevant gene fusions within the MAPK pathway might have been undetected in some cases by the methods applied. Therefore, some MAPK altered cases might have been misclassified as MAPK wildtype by the methods applied, which is a shortcoming of the present study.

Phosphorylation status of ERK was used within the PTT2.0 registry as a general marker for MAPK pathway activation. Although we found overall a significantly higher pERK level in genomically MAPK-altered samples, the power of high pERK to predict a genomic MAPK alterations was low (sensitivity 58.3%). A disconnection between the presence of a MAPK alteration and the level of pERK H-score became particularly evident in the analysis of subgroups of the cohort, which revealed that pLGG, the entity with highest abundance of MAPK alterations, and EPN, where MAPK alterations were as expected absent^1^, had the highest and statistically indifferent mean pERK H-scores of the cohort. In line with our results, the discrepancy between mutational status and pERK-level has been described in the literature before. pERK positivity was shown not to correlate with the mutational status of NRAS and/or BRAF in melanoma in adult patients^14^, suggesting, that also in cancer cells without activating MAPK alterations high levels of MAPK activity may be present. Wagle et al. used a gene expression score including 10 genes (MAPK activity score; MPAS) to determine MAPK pathway activation in several adult cancer types^15^. MAPK pathway activation was highest in melanoma, however the activity level was independent of MAPK-mutational status, indicating an entity specific and not necessarily mutation-dependent activation of the pathway. Moreover, the sensitivity towards MAPK-targeting MEK inhibitor treatment was highest in melanoma independent of the presence or absence of genetic MAPK alterations, indicating a disconnection between mutation status and the activity of the MAPK pathway as well as the response to MAPK targeted therapy. We have recently shown, that MAPK inhibitor sensitivity gene expression scores (MSS) can predict sensitivity to MAPKi-classes in pediatric tumors^16^. Notably, the highest MSS were obtained in pLGG that are typically enriched for activating MAPK alterations. EPN, the group without MAPK alterations but high mean pERK in the present cohort, showed intermediate mean MSS indicating a certain degree of sensitivity and supporting the hypothesis of MAPK alteration independent MAPK pathway activation. In line with this, alternative mechanisms of MAPK activation are described for EPN e.g. on gene expression level (especially posteriorfossa group A (PFA) which were the largest molecular subgroup of our cohort (*n* = 20 PFA of 30 EPN cases)^17,18^. Of note, the PTT2.0 cohort consists of progressed/relapsed tumors and part of the samples analyzed were exposed to prior antineoplastic treatment, which also might have an effect on pERK expression independent of MAPK alterations^19,20^. As stated above, PTT2.0 used pERK H-score as general marker of MAPK activity. However, parts of our analyses question the validity of the single marker pERK as sufficient and reliable activation parameter. We found overall low levels of pERK in all samples with *BRAF V600E* mutations (lower median and mean compared to the whole cohort) indicating a low MAPK pathway activity in *BRAF V600E* background. Of note, only one of all 24 tumor samples with genomic MAPK alteration and none of the five *BRAF V600E* mutated tumors had been treated with MAPK directed targeted agents before surgery, which excludes an extrinsic therapy induced reduction of pERK levels in these cases. Moreover, pERK H-scores were significantly lower in *BRAF V600E* compared to samples with *KIAA1549::BRAF* fusion. This result stands in contrast to several previous observations: *BRAF V600E* is a strong inducer of the MAPK pathway and has a 500-fold kinase activity compared to BRAF wild type^21^. MPAS analysis in a large cohort of pLGG samples revealed that *BRAF V600E* positive samples had a significantly higher activation level of the MAPK pathway compared to samples with *KIAA1549::BRAF* fusions^16^. Finally, response of *BRAF V600E* positive pLGG tumors led to approval of the MAPKi combination therapy dabrafenib/trametinib, highlighting the relevant pathway activation in these tumors^2^. A possible explanation for the failure of pERK to correctly predict MAPK activity in this context might be that the phosphorylation status of ERK is highly dependent on feedback mechanisms within the MAPK-pathway. The BRAF V600E kinase is permanently active but irresponsive to negative feedback. This leads on the one hand to high levels of pMEK and on the other hand increased DUSP6 expression. Activation of ERK caused by high levels of pMEK and DUSP6 induced deactivation of ERK lead to an overall steady-state level of pERK^22^. This might explain, why we did not find pERK increased in BRAF V600E samples despite an activated pathway, which impairs the robustness of pERK as a marker for MAPK pathway activation in this genetic background.

Taken together, pERK H-score and the presence of genetic MAPK alterations are not strongly associated in pediatric cancer. Entities with high pERK levels but missing genomic MAPK alterations support the theory that MAPK activation can occur in pediatric cancer without detected MAPK alterations on genomic level like it was described for adult populations. On the other hand, cases with low pERK in the presence of a known MAPK activating genomic alteration challenge the validity of pERK as a single and reliable marker for MAPK pathway activity. In the future, gene expression-based scores like MPAS or MSS should be implemented in the diagnostic work up of pediatric tumors to inform physicians more reliably about MAPK activation and potential use of MAPKi in cases where tumors lack response-predicting MAPK-alterations.

## Data Availability

The dataset used and analyzed during the current study is available from the corresponding author upon reasonable request.
